# PARP1 Upregulation in Recurrent Oral Cancer and Treatment Resistance

**DOI:** 10.3389/fcell.2021.804962

**Published:** 2022-01-05

**Authors:** Feifei Wang, Odjo G. Gouttia, Ling Wang, Aimin Peng

**Affiliations:** Department of Oral Biology, University of Nebraska Medical Center, Lincoln, NE, United States

**Keywords:** PARP inhibition, oral cancer, DNA repair, chemotherapy, PARP1

## Abstract

First-line treatments for oral cancer typically include surgery, radiation, and in some cases, chemotherapy. Radiation and oral cancer chemotherapeutics confer cytotoxicity largely by inducing DNA damage, underscoring the importance of the cellular DNA damage repair and response pathways in cancer therapy. However, tumor recurrence and acquired resistance, following the initial response to treatment, remains as a major clinical challenge. By analyzing oral tumor cells derived from the primary and recurrent tumors of the same patient, our study revealed upregulated PARP1 expression in the recurrent tumor cells. Cisplatin and 5-fluorouracil treatment further augmented PARP1 expression in the recurrent, but not the primary, tumor cells. Post-treatment upregulation of PARP1 was dependent on the catalytic activities of PARP and CDK7. Consistent with the established function of PARP1 in DNA repair, we showed that overexpression of PARP1 rendered the primary tumor cells highly resistant to DNA damage treatment. Conversely, PARP inhibition partially reversed the treatment resistance in the recurrent tumor cells; combinatorial treatment using a PARP inhibitor and cisplatin/5-fluorouracil significantly sensitized the tumor response *in vivo*. Taken together, we reported here PARP1 upregulation as a clinically relevant mechanism involved in oral cancer recurrence, and suggested the clinical benefit of PARP inhibitors, currently approved for the treatment of several other types of cancer, in oral cancer.

## Introduction

Oral cancer, including cancers of the mouth and the back of the throat, is the sixth most common cancer worldwide. In the United States, approximately 50,000 new oral cancer cases are diagnosed each year. First-line treatments for oral cancer typically include surgery and radiation, with chemotherapy added to decrease the possibility of metastasis, to eliminate residual tumor cells after surgery, to enhance the efficacy of radiation, and for patients with confirmed distant metastasis (Casiglia and Woo, 2001; Gau et al., 2019; Johnson et al., 2020). Radiation and oral cancer chemotherapeutics, such as cisplatin and 5-fluorouracil (5-FU), confer cytotoxicity largely by inducing DNA damage. Oral cancer caused by HPV generally responds to the existing treatments, with over 80% 5-year survival rate for stage III and IV patients. On the other hand, only 10–20% HPV- oral cancer patients at stage III and IV survive the 5-year period. Moreover, the survival rate of oral cancer has not improved significantly over the past decades. Thus, it is important, and urgent, to discover new mechanisms of treatment resistance, and to develop new therapeutics and combinations to overcome resistance in oral cancer.

The cellular DNA damage response (DDR) pathway plays a crucial role in determining the treatment outcome of radiation and genotoxic chemotherapeutics (Jackson and Bartek, 2009). The DDR encompasses complex signaling pathways that lead to cell cycle arrest and cell death. On the other hand, the DDR employs various DNA repair mechanisms to remove DNA damage, and promote cell survival (Ciccia and Elledge, 2010). Based on these principles, it has been long proposed that targeting certain elements of the DDR can effectively sensitize tumor cells to radiation and other DNA damaging drug treatments (Zhou et al., 2003; Liang et al., 2009; Jalal et al., 2011; Lord and Ashworth, 2012).

Among the most promising new anti-cancer targets are poly (ADP-ribose) polymerases (PARPs). PARPs catalyze the attachment of poly (ADP-ribose) chains to substrate proteins, a process termed PARylation (Luo and Kraus, 2012; Dulaney et al., 2017; Martin-Hernandez et al., 2017; Ray Chaudhuri and Nussenzweig, 2017). In particular, PARP1 accounts for over 90% of DNA damage-induced PARylation, thereby playing an important role in the DDR. PARP1 acts as an early and upstream sensor for a variety of DNA damage, and is required for the recruitment of many downstream repair factors, such as X-ray repair cross-complementing protein 1 (XRCC1) (Luo and Kraus, 2012; Dulaney et al., 2017; Martin-Hernandez et al., 2017; Ray Chaudhuri and Nussenzweig, 2017). Consistent with the function of PARP1 in DNA repair, its inhibition has been considered as a valid approach to enhance the cytotoxic effect of radiation and chemotherapeutics, as well as to exploit synthetic lethality in tumors with defective DSB repair (Dulaney et al., 2017; Lord and Ashworth, 2017). Olaparib, a PARP inhibitor (PARPi), was approved by FDA and EMA in 2014 for the treatment of ovarian cancer with BRCA1 and BRCA2 mutations. The approval was extended also to breast cancer in 2018, and to prostate and pancreatic cancer in 2019. With these emerging successes of PARPi, it is important to investigate the involvement of PARPs in the pathophysiology of oral cancer, and to evaluate the potential application of PARPi in the treatment of oral tumors, particularly those exhibiting resistance to DNA damaging agents. In this study, we revealed upregulation of PARP1 as a mechanism that rendered oral cancer cells resistant to treatment, and PARPi as effective agents that re-sensitized these cells to chemotherapy *in vitro* and *in vivo*.

## Results

### Upregulation of PARP1 in the Recurrent Oral Tumor Cells

**FIGURE 1:**
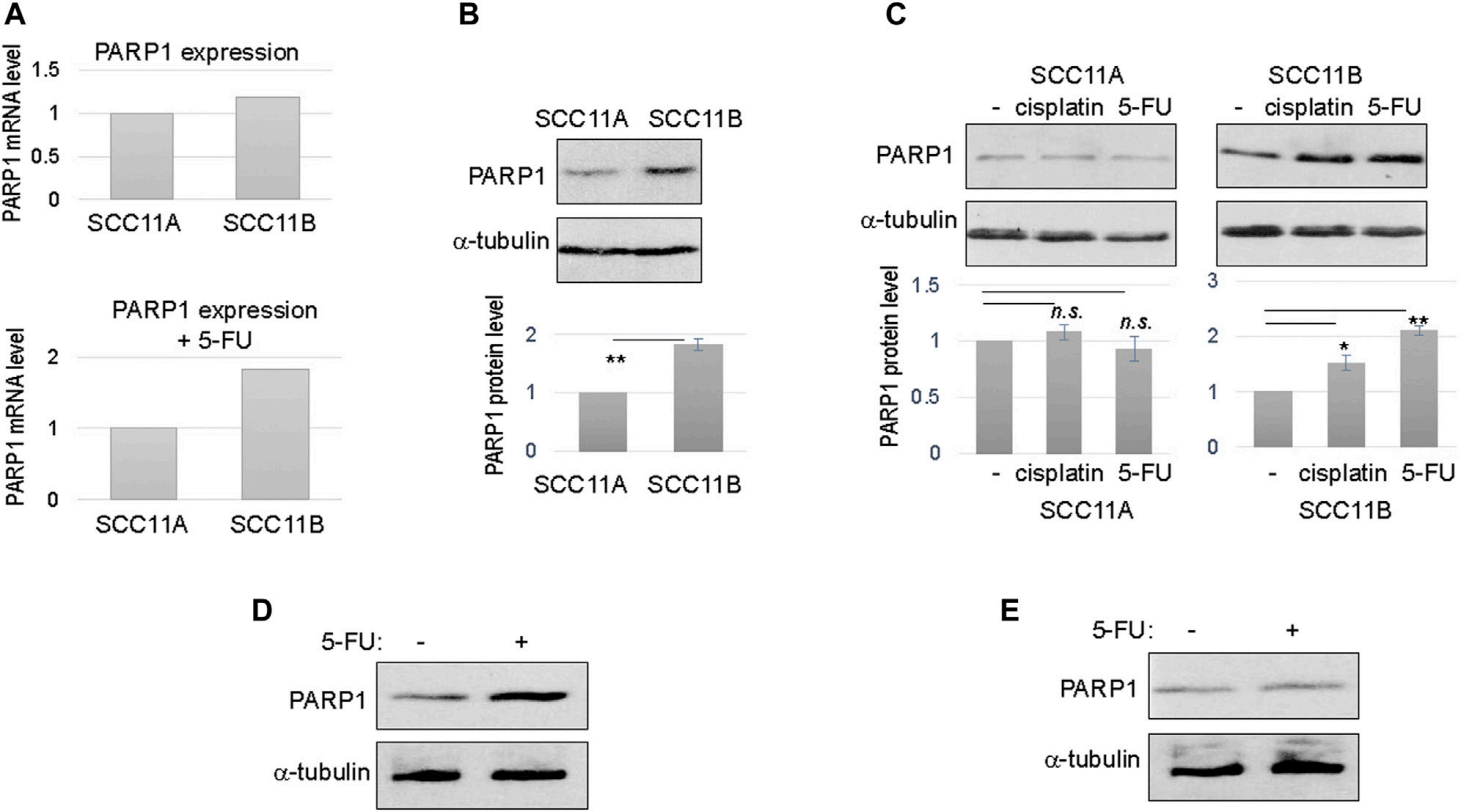
Upregulation of PARP1 in recurrent oral cancer cells. **(A)** The levels of PARP1 mRNA in SCC11A and SCC11B cells, without DNA damage **(upper panel)**, or with 5-FU treatment **(lower panel)**. **(B)** SCC11A and SCC11B cells were analyzed by immunoblotting for the protein levels of PARP1 and *α*-tubulin (loading control). The band intensity was quantified, and the PARP1 to *α*-tubulin ratio was shown in the lower panel. The relative PARP1 protein level in SCC11B was normalized to that in SCC11A. The average of three independent experiments was shown, with statistical analysis (**p < 0.01). **(C)** SCC11A and SCC11B cells were treated with cisplatin (10 μM) or 5-FU (10 μM), as indicated, for 1 day. The treated and untreated cells were analyzed by immunoblotting for PARP1 and *α*-tubulin. The band intensity was quantified, and the PARP1 to *α*-tubulin ratio was shown in the lower panel. The relative PARP1 protein level in drug treated cells was normalized to that in untreated cells. The average of three independent experiments was shown, with statistical analysis (*p < 0.05, n.s. not significant). **(D)** SCC10B cells were treated with or without 5-FU (10 μM), for 1 day. The cells were then analyzed by immunoblotting for PARP1 and *α*-tubulin. **(E)** The oral mucosal epithelial cells were treated with or without 5-FU (10 μM), for 1 day. The cells were then analyzed by immunoblotting for PARP1 and *α*-tubulin.

To shed new light on oral cancer resistance and recurrence, we obtained a pair of patient-derived, matched, oral cancer cell lines. Of these lines, SCC11A was established from the initial oral tumor, and SCC11B was obtained from the recurrent tumor after treatment with radiation and chemotherapy. The patient expired due to tumor recurrence and the subsequent metastasis. These matched cell lines offer a physiologically relevant model to study molecular events that underlie treatment evasion and tumor recurrence. Interestingly, RNA sequencing analysis of gene expression revealed that SCC11B exhibited an elevated RNA level of PARP1, particularly when cells were treated with 5-FU (Figure 1A).

To confirm the RNA sequencing results, we analyzed the protein level of PARP1 in SCC11A and SCC11B cells. In fact, PARP1 protein in SCC11B was approximately two fold more abundant than that in SCC11A (Figure 1B). Upon treatment with cisplatin and 5-FU, two chemotherapeutic drugs used for oral cancer, PARP1 expression was further increased in SCC11B, by approximately 50 and 100%, respectively (Figure 1C). By comparison, cisplatin and 5-FU did not induce PARP1 expression in SCC11A cells (Figure 1C).

Furthermore, we noted a similar fashion of 5-FU-induced PARP1 upregulation in SCC10B, another oral cancer cell line derived from recurrent tumor (Figure 1D). PARP1 expression was unchanged in the control, oral mucosal epithelial cells upon 5-FU treatment (Figure 1E), confirming that upregulation of PARP1 reflects an acquired, post-treatment mechanism in some tumor cells.

### The Catalytic Activity of PARP1 Mediates its Own Upregulation After DNA Damage

**FIGURE 2:**
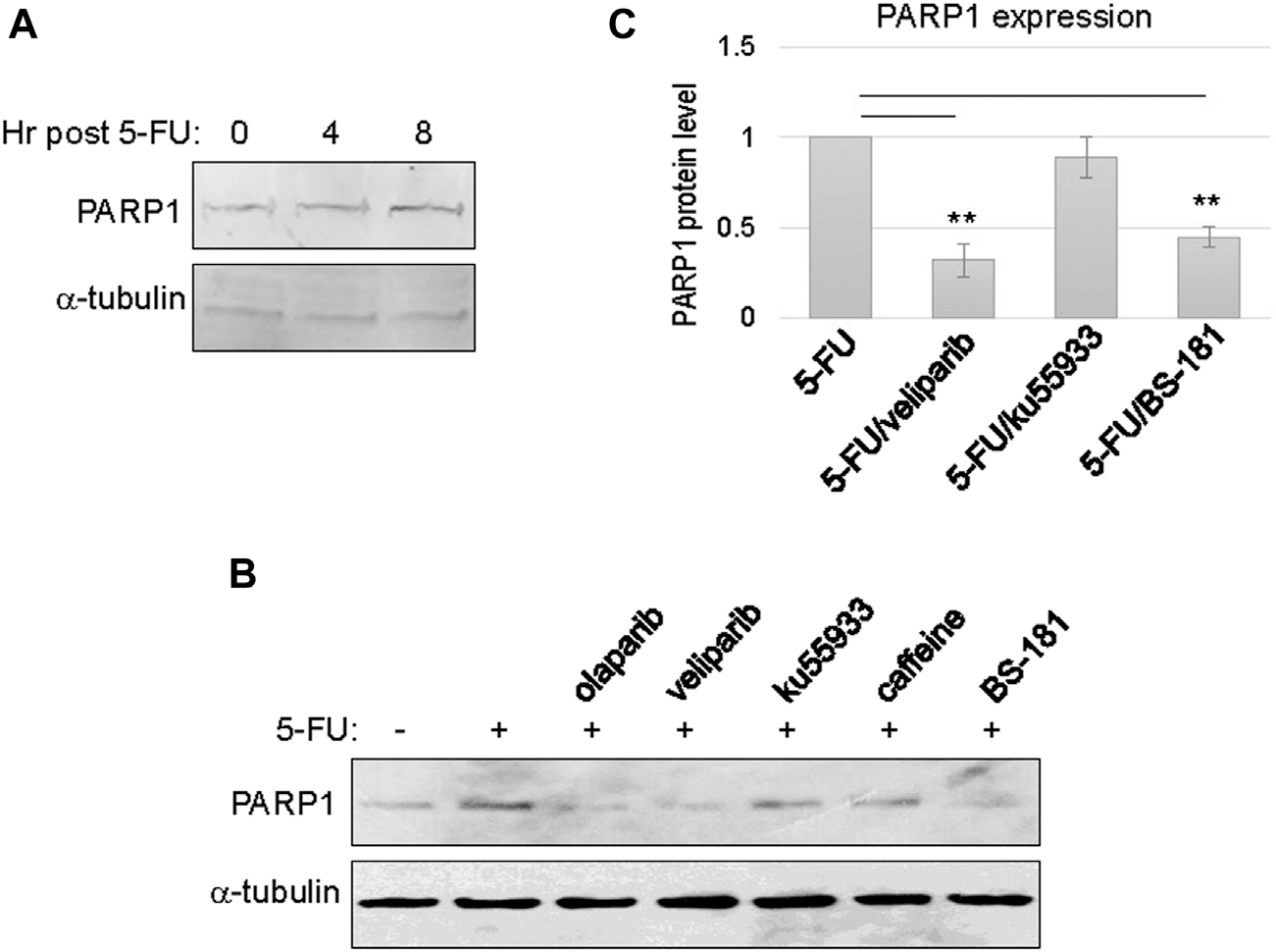
PARP1 upregulation upon chemotherapeutic treatments in recurrent oral tumor cells. **(A)** SCC11B cells were treated with or without 5-FU (10 μM), as indicated, for 4 or 8 h. The treated and untreated cells were analyzed by immunoblotting for PARP1 and *α*-tubulin. **(B)** SCC11B cells were treated with or without 5-FU (10 μM), in combination with olaparib (10 μM), veliparib (10 μM), Ku55933 (10 μM), caffeine (3 mM), and BS-181 (1 μM), as indicated, for 1 day. The treated and untreated cells were analyzed by immunoblotting for PARP1 and *α*-tubulin. **(C)** Quantification of PARP1 expression in panel **(B)**. The relative PARP1 protein level in cells with combination drug treatments was normalized to that in cells treated with 5-FU alone. The average of three independent experiments was shown, with statistical analysis.

Upregulation of PARP1 expression was observed between 4 and 8 h post 5-FU treatment, possibly reflecting the time frame of PARP1 transcription and translation (Figure 2A). Next, we sought to reveal more mechanistic insights into the cellular activities that govern PARP1 upregulation. Interestingly, inhibition of PARP *per se*, using olaparib or veliparib, prevented PARP1 upregulation in the presence of 5-FU treatment (Figures 2B,C). By comparison, inhibition of ATM with ku55933, or ATM/ATR with caffeine, did not significantly alter PARP1 upregulation (Figures 2B,C). These results indicated that DNA damage-induced PARP1 upregulation does not rely on the conventional signaling pathway initiated via ATM/ATR activation. Moreover, a selective inhibitor of CDK7, BS-181, also reduced PARP1 expression (Figures 2B,C).

### PARP1 Upregulation Renders Oral Cancer Cells Resistant to DNA Damaging Drugs

**FIGURE 3:**
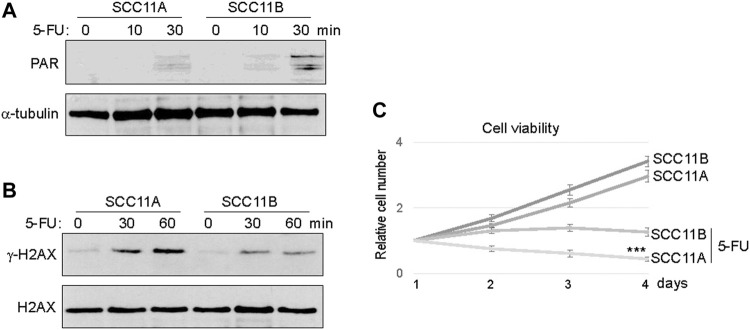
SCC11B cells exhibited increased PARylation and cell resistance. **(A)** SCC11A and SCC11B cells were incubated in 5-FU (10 μM), for 10 or 30 min, as indicated. Cells were harvested for immunoblotting. **(B)** SCC11A and SCC11B cells were incubated in 5-FU (10 μM), for 30 or 60 min, as indicated. Cells were harvested for immunoblotting. **(C)** Cell viability assay was performed as in the Materials and Methods. SCC11A and SCC11B cells were incubated for 4 days. 5-FU (10 μM) was added at day 1. The cell numbers at days 2–4 were normalized to that at day 1 (untreated). The mean values and standard derivations, from three independent experiments, were shown.

Consistent with PARP1 upregulation, an elevated level of Poly (ADP-ribosyl)ation (PARylation) was induced in SCC11B cells, compared to SCC11A cells, upon 5-FU treatment (Figure 3A). On the other hand, accumulation of *γ*-H2AX appeared alleviated in SCC11B cells, potentially owing to PARP1-mediated DNA repair (Figure 3B). Using a cell viability assay, we confirmed that SCC11B cells exhibited increased resistance to 5-FU treatment (Figure 3C). Thus, PARP1 upregulation during oral tumor recurrence correlated with increased PARylation, decreased DNA damage accumulation, and acquired drug resistance.

**FIGURE 4:**
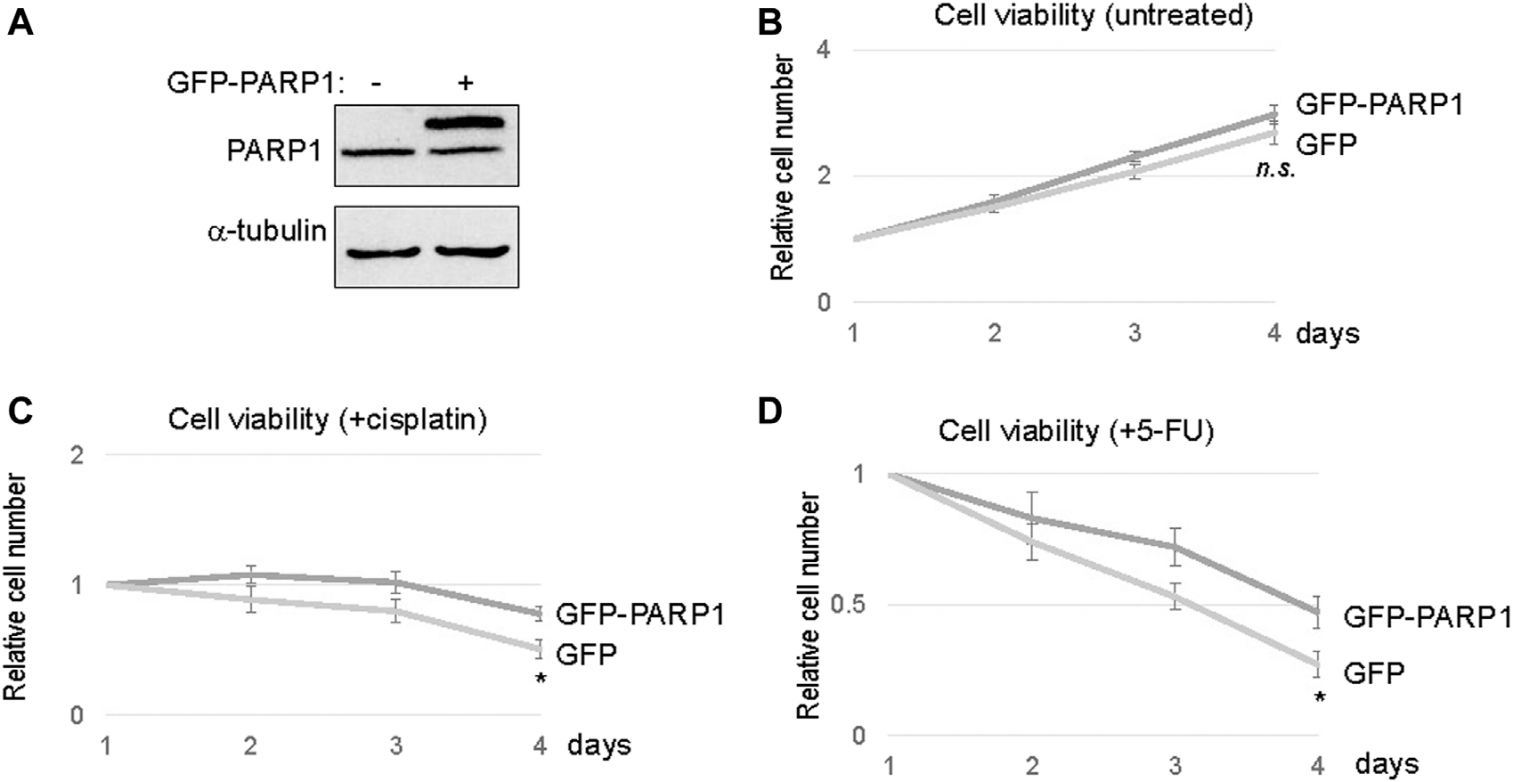
PARP1 upregulation rendered SCC11A cells more resistant to chemotherapeutic drugs. **(A)** The expression of recombinant and endogenous PARP1 was shown by immunoblotting. **(B)** SCC11A cells expressing GFP-PARP1 or control GFP vector were incubated for 4 days. Cell viability was determined as described in Materials and Methods, the cell numbers at days 2–4 were normalized to that at day 1. The mean values and standard derivations, from three independent experiments, were shown. **(C)** SCC11A cells expressing GFP-PARP1 or control GFP vector were incubated for 4 days. Cisplatin (10 μM) was added at day 1. The cell numbers at days 2–4 were normalized to that at day 1 (untreated). The mean values and standard derivations, from three independent experiments, were shown. **(D)** SCC11A cells expressing GFP-PARP1 or control GFP vector were incubated for 4 days. 5-FU (10 μM) was added at day 1. The cell numbers at days 2–4 were normalized to that at day 1 (untreated). The mean values and standard derivations, from three independent experiments, were shown.

To directly assess the functional impact of PARP1 upregulation, we expressed recombinant PARP1 in SCC11A cells, to approximately two to three fold over the endogenous level (Figure 4A). Compared to the vector control, PARP1 expression alone did not markedly influence the cell viability (Figure 4B). However, significant proliferative advantages were observed in PAPR1-expressing cells upon treatment with cisplatin or 5-FU (Figures 4C,D). Together, these results indicated that upregulation of PARP1 in SCC11A cells adequately conferred tumor cell resistance to chemotherapeutic drugs.

### PARP1 Targeting in Recurrent Oral Cancer Cells and Tumors

**FIGURE 5:**
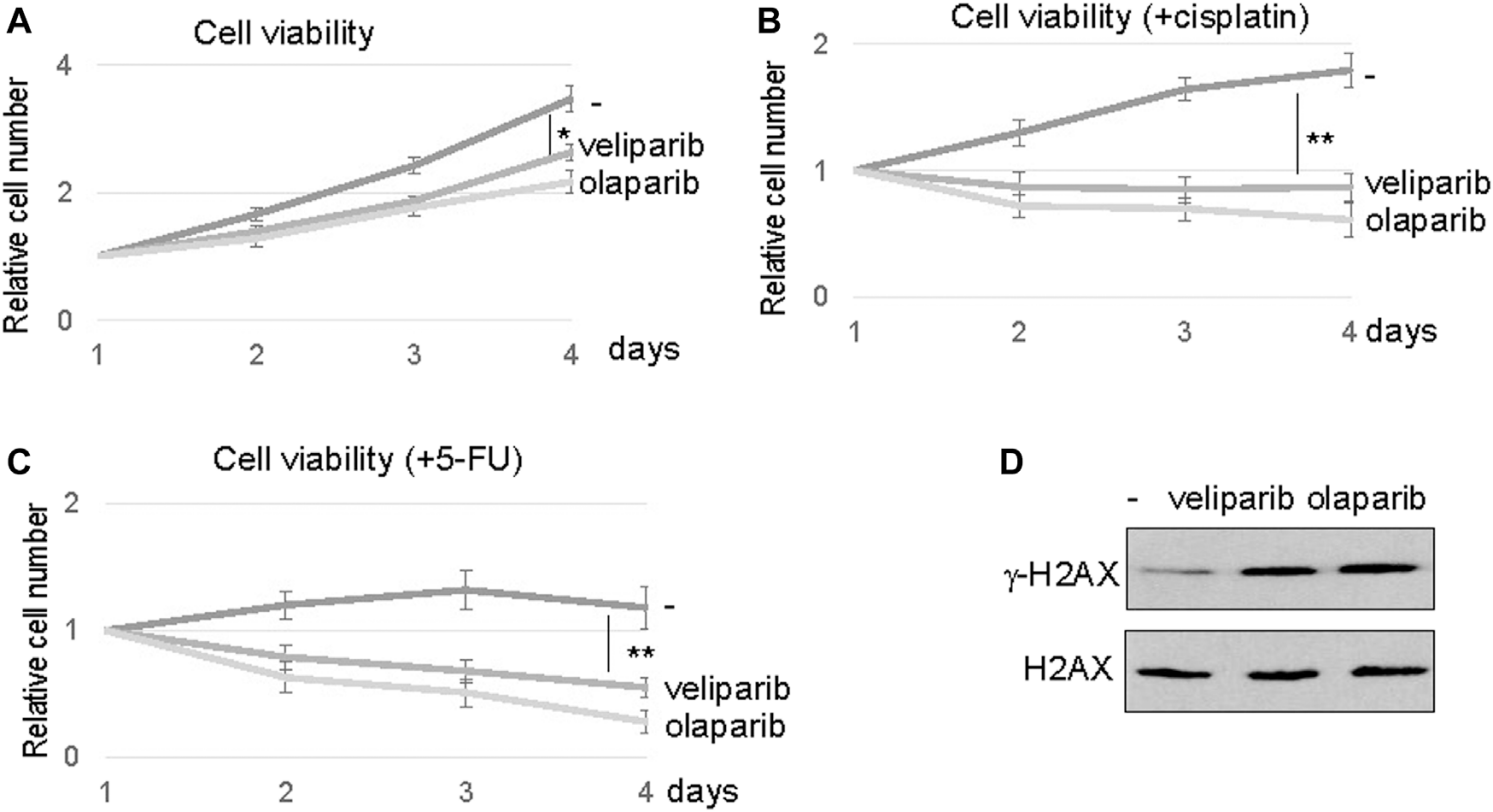
PARP inhibition sensitized SCC11B cells to DNA damage. **(A)** SCC11B cells were incubated for 4 days, with or without veliparib and olaparib, as indicated. The cell numbers at days 2–4 were normalized to that at day 1 (untreated). The mean values and standard derivations, from three independent experiments, were shown. **(B)** SCC11B cells were incubated for 4 days, with or without veliparib and olaparib, as indicated. Cisplatin (10 μM) was added at day 1. The cell numbers at days 2–4 were normalized to that at day 1 (untreated). The mean values and standard derivations, from three independent experiments, were shown. **(C)** SCC11B cells were incubated for 4 days, with or without veliparib and olaparib, as indicated. 5-FU (10 μM) was added at day 1. The cell numbers at days 2–4 were normalized to that at day 1 (untreated). The mean values and standard derivations, from three independent experiments, were shown. **(D)** SCC11B cells were treated with or without veliparib and olaparib for 1 day. The cells were harvested and analyzed by immunoblotting for *γ*-H2AX and H2AX.

Our findings prompted us to evaluate the potential of PARP1 targeting in enhancing the therapeutic response of oral tumor cells, particularly in SCC11B cells that exhibited PARP1 upregulation. PARP inhibition using two clinically approved inhibitors, veliparib and olaparib, reduced the viability of SCC11B cells, suggesting the possible use of PARPi as monotherapeutic agents (Figure 5A). However, a more profound therapeutic benefit was observed, when SCC11B cells were treated with PARPi in combination with cisplatin or 5-FU (Figures 5B,C). In these cases, both veliparib and olaparib elicited synergistic effects with cisplatin and 5-FU. Interestingly, veliparib and olaparib increased the induction of *γ*-H2AX after 5-FU treatment (Figure 5D). This finding indicated that inhibition of PARP1 caused increased accumulation of DNA damage, particularly DNA double strand breaks, after therapeutic exposure to 5-FU.

**FIGURE 6:**
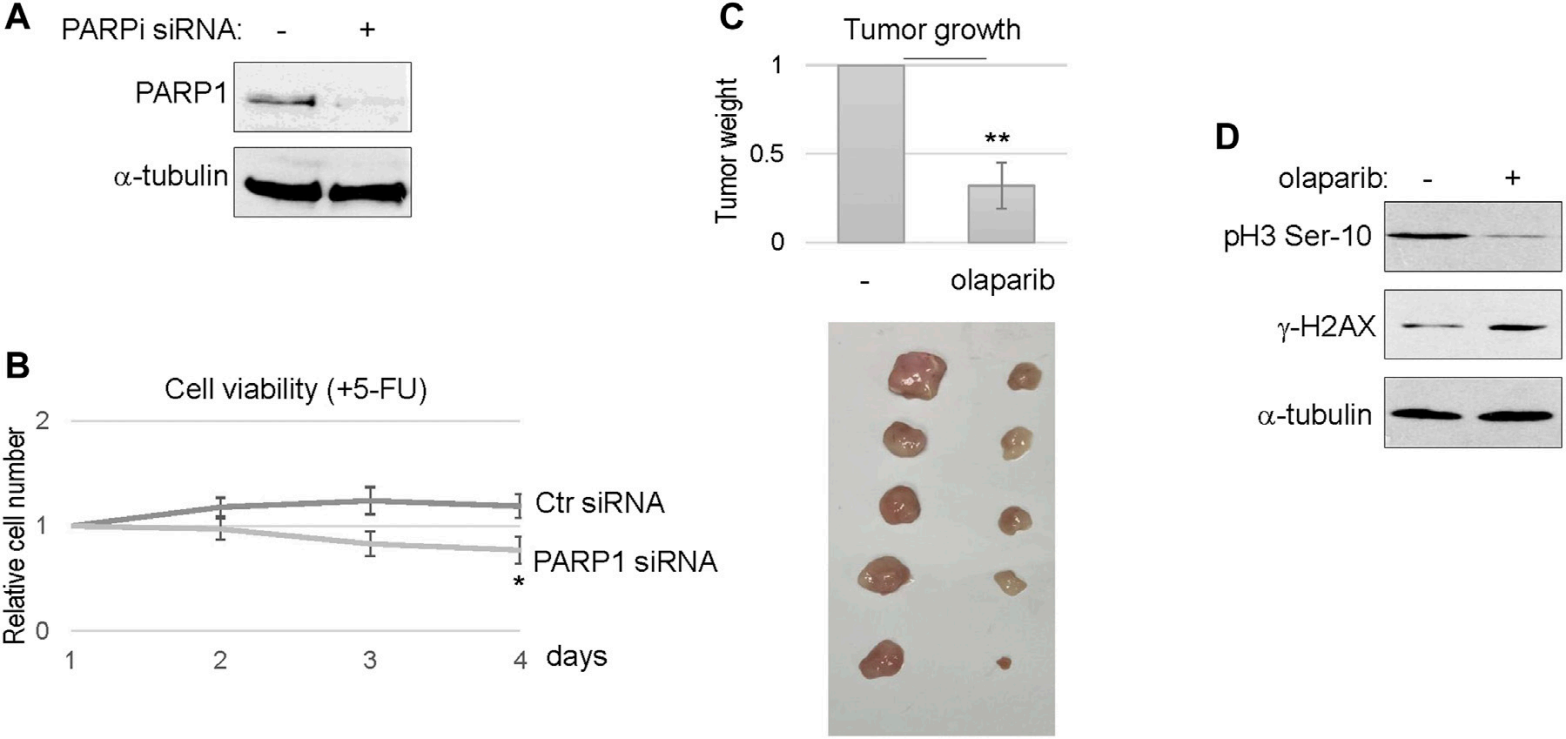
PARP targeting sensitized the SCC11B tumor response to chemotherapy. **(A)** SCC11B cells were treated with PARP1 siRNA or control siRNA, as described in Materials and Methods. Cells were analyzed by immunoblotting to confirm PARP1 depletion. **(B)** SCC11B cells were incubated for 4 days, with control or PARP1 siRNA, as indicated. 5-FU (10 μM) was added at day 1. The cell numbers at days 2–4 were normalized to that at day 1 (untreated). The mean values and standard derivations, from three independent experiments, were shown. **(C)** SCC11B cells were implanted into immunodeficient mice to form tumors. As described in Materials and Methods, mice were then treated with cisplatin/5-FU, with or without olaparib. Tumors were excised, and shown in the lower panel. The average tumor weight of olaparib/cisplatin/5-FU-treated group was normalized to that of cisplatin/5-FU. The mean values and standard derivations were shown, statistical significance was determined by Student’s t-test (*n* = 5 per group). **(D)** Tumor samples were processed, as described in Materials and Methods, and analyzed by immunoblotting.

We further confirmed the efficacy of PARP1 targeting using siRNA-mediated PARP1 depletion (Figure 6A). Consistent with PARP inhibition, reducing the expression level of PARP1 in SCC11B cells enhanced the therapeutic outcome of 5-FU, as indicated by decreased cell viability (Figure 6B). Finally, we established SCC11B xenograft tumor models in immunodeficient mice, to evaluate the effect of PARPi in chemotherapy. A combination regimen with both cisplatin and 5-FU was used, as in the clinical treatment of head and neck and many other cancers. Compared to chemotherapy alone, combination with olaparib substantially improved the tumor responses, and deceased the final tumor volume by approximately three fold (Figure 6C). Biochemical analyses of the tumor samples confirmed that PARPi treatment increased DNA damage accumulation, and decreased cell proliferation, as judged by *γ*-H2AX and phospho-histone H3, respectively (Figure 6D).

## Materials and Methods

### Antibodies and Chemicals

Sodium dodecyl sulfate-polyacrylamide gel electrophoresis (SDS-PAGE) and immunoblotting was performed as described previously (Wang et al., 2019a). Anti-PARP1, H2AX, Histone H3 Ser-10, and *γ*-H2AX antibodies were purchased from Cell Signaling (Danvers, MA); anti-poly (ADP-ribose) polymer antibody was obtained from Santa Cruz Biotechnology (Dallas, TX); *α*-tubulin antibody was a gift from Dr. James Wahl (University of Nebraska Medical Center). The intensity of band signals was measured using NIH ImageJ software. Cisplatin and 5-fluorouracil were purchased from Sigma (St. Louis, MO); olaparib and veliparib were obtained from Selleckchem (Houston, TX) and Santa Cruz Biotech (Dallas, TX), respectively.

### Cell Culture and Analyses

Human oral squamous-cell carcinoma cell lines SCC11A (UM-SCC-11A), SCC11B (UM-SCC-11B), and SCC10B (UM-SCC-10B) were obtained from the University of Michigan, and characterized genetically and morphologically (Brenner et al., 2010; Wang et al., 2012; Luong et al., 2016). These cells were maintained in Dulbecco’s modified Eagle medium (DMEM, Sigma) supplemented with 10% fetal bovine serum (FBS, Sigma). Mouse oral mucosal epithelial cells were purchased from Cell Biologics (Chicago, IL), and maintained in the recommended epithelial medium (Cell Biologics). To measure SCC11A and SCC11B cell sensitivity to cisplatin and 5-FU, cells were treated with cisplatin at indicated concentrations, and incubated for 1–4 days. The numbers of viable cells were counted using a hemocytometer. GFP-PARP1 was described in our previous study (Wang et al., 2019b), and was transfected to SCC11A cells using lipofectamine (Thermo Fisher Scientific, Waltham, MA). Cell pellets were submitted to Genewiz (South Plainfield, NJ) for RNA sequencing analysis. PARP1 siRNA (target sequence UGA​CUU​GGA​AGU​GAU​CGA) were purchased from Integrated DNA Technologies (IDT), and transfected into cells using Lipofectamine RNAiMAX (Thermo) following the protocol recommended by the manufacturer. A non-targeting control, or scramble siRNA was used as a control.

### Mouse Tumor Studies

Athymic nude mice were purchased from the Jackson Laboratory (Bar Harbor, ME) and housed at the animal facility at the UNMC College of Dentistry. SCC11B cells were implanted into 6-week old mice by a single subcutaneous injection of tumor cells (2–6 × 10^5^ cells in 100 μl of sterile PBS). To test how tumors respond to chemotherapy, once the tumor size reached 50 mm^3^, cisplatin and 5-FU (5 mg/kg mouse), with or without olaparib (10 mg/kg mouse) were administered intraperitoneally on days 1 and 3. Ten days after the initial treatment, the mice were euthanized, and tumors were removed and weighed.

To prepare tumor lysate for immunoblotting analysis, excised tumor samples were frozen on dry ice, and cut into small pieces. 20 μl/mg of RIPA (20 mM Tris-Cl (pH 7.4) 1 mM EDTA. 0.5 mM EGTA. 1% Triton X-100. 0.1% sodium deoxycholate. 0.1% SDS. 150 mM NaCl) was added, and the samples were homogenized. The samples were then centrifuged, and supernatants were collected for immunoblotting.

### Statistical Analysis

Statistical analyses were performed in cell viability assays and in the tumor weight measurements. Briefly, data were analyzed using an unpaired 2-tailed Student’s t test to determine the statistical significance. A *p*-value less than 0.05 is considered as significant.

## Discussion

### Acquired Cancer Resistance in Oral Cancer Is Associated With Altered DDR Pathways

In this study, we reported PARP1 upregulation during the recurrence of oral tumor, using patient-derived cell lines. We showed that the elevated PARP1 expression conferred treatment resistance in the primary oral tumor cells, and that the recurrent tumor cells are highly dependent on PARP activity for treatment evasion. Presumably, the initial clinical treatment using radiation and chemotherapy selected for cells with PARP1 upregulation. Furthermore, in these recurrent oral tumor cells, cisplatin and 5-fluorouracil were capable of inducing the gene expression of PARP1. To our knowledge, this mechanism of treatment-induced PARP1 expression is new. Strikingly, this phenomenon was not seen in the matched primary tumor cells, or in a control oral mucosal epithelial cell line, pointing to specific dysregulation of PARP1 expression that was acquired during tumor recurrence.

Our findings add to the emerging understanding of how tumor resistance and recurrence is driven by specific alterations of the DDR. Deficient DNA damaging signaling, particularly the ATM kinase-mediated pathway, has been observed in oral cancer cells, in correlation with reduced responsiveness to cisplatin (Wang et al., 2012). Other studies revealed polymorphisms of DDR genes as potential risk factors that promote head and neck cancer progression. Altered expression levels of DNA repair genes, including both upregulation and downregulation, have been shown in oral cancer studies (Wang et al., 2007; Jenkins et al., 20132013; Ali et al., 2017; Dylawerska et al., 2017; Psyrri et al., 2021). Thus, detailed functional studies are necessary to further elucidate how these DDR alterations impact the progression and treatment responses of oral cancer.

### PARP1 as an Anti-Cancer Drug Target in Oral Cancer

With the proven clinical benefits of PARPi in other solid tumors, the potential application of PARPi in oral cancer treatment has been enthusiastically proposed (Glorieux et al., 2017; Moutafi et al., 2021). This therapeutic idea was further supported by multiple lines of preclinical studies. For example, PARPi was found effective in head and neck cancer with SMAD4-deficiency (Hernandez et al., 2020). The efficacy of PARPi, in combination with radiation, platinum-based drugs, DNA-PKcs inhibitor, PD-1/PD-L1 blockage agents, and many other drugs, has been suggested (Glorieux et al., 2017; Moutafi et al., 2021). Building on these premises, multiple ongoing clinical trials are evaluating the efficacy of PARPi in monotherapy or combination therapy of oral cancer. Combinatorial treatments using PARPi and conventional chemotherapy attracted particular interests, as potentially promising opportunities to overcome tumor resistance to either PARPi or chemotherapy alone (Lu et al., 2018; Li et al., 2020; McMullen et al., 2020).

Evidence provided in this study supported the use of PARPi in oral cancer therapy, especially in combination with cisplatin or 5-fluorouracil. PARP1 suppressed the induction of DNA double strand breaks following cisplatin or 5-fluorouracil treatment. This is well in line with the role of PARP1 in single strand break repair, and consistent with the observation of increased cell resistance upon PARP1 expression.

### PARP1 Is Upregulated Upon DNA Damage Treatment in Resistant Oral Cancer Cells

Our study revealed upregulation of PARP1 expression in at least some recurrent oral tumor cells, as one of the underlying mechanisms of treatment resistance and tumor recurrence. Thus, the physiological relevance of PARP1 in oral cancer recurrence provides an additional rationale for PARP1 targeting. Unlike the primary oral tumor cells, recurrent tumor cells gained the capability of inducing PARP1 expression upon cisplatin or 5-fluorouracil treatment. This treatment-induced PARP1 expression can potentially serve as a prognostic biomarker that predicts both tumor resistance to DNA damaging agents, and therapeutic benefits of PARPi in combination therapy.

Regulation of PARP1 gene expression remains to be better understood. DNA damage-induced PARP1 expression, as shown in our studies, was not dependent on ATM/ATR kinase activities. PARP1 upregulation was disrupted by inhibitors of CDK7 and PARP. CDK7 is known to be associated with, and phosphorylate, transcription factors (Fisher, 2019); existing evidence also supported a role of PARP1 in transcriptional regulation (Schiewer and Knudsen, 2014). Interestingly, previous studies of mouse PARP1 expression suggested an autoregulatory model in which PARP1 binds to its own promoter region, and suppresses transcription (Vidaković et al., 2009). Thus, it shall be investigated if PARPi influences its own expression by trapping PARP1 in its promoter region, or through additional transcriptional factors that are directly or indirectly modulated through PARylation.

Taken together, our studies reported PARP1 upregulation as a clinically relevant mechanism of tumor resistance, and suggested PARPi as promising therapeutic intervention, in combination with chemotherapy. Further delineation of the underlying mechanisms will potentially shed new light on the signaling network of tumor recurrence, and uncover additional drug targets to cripple cancer resistance.

## Data Availability

The raw data supporting the conclusion of this article will be made available by the authors, without undue reservation.
